# Assessment of Financial Impact of Expanding the Scope of Drug Usage in South Korea

**DOI:** 10.34172/ijhpm.8812

**Published:** 2025-07-13

**Authors:** Su-Yeon Yu, Dong-Sook Kim, Hyungmin Kim, Junwoo Jo, Hyunduck Kim, Euna Han

**Affiliations:** ^1^College of Pharmacy, Kangwon National University, Chuncheon, Republic of Korea.; ^2^Department of Health Administration, College of Nursing and Health, Kongju National University, Kongju, Republic of Korea.; ^3^Department of Drug Management, National Health Insurance Service, Wonju, Republic of Korea.; ^4^College of Pharmacy, Seoul National University, Seoul, Republic of Korea.; ^5^Department of Statistics, Kyungpook National University, Daegu, Republic of Korea.; ^6^College of Pharmacy, Yonsei Institute of Pharmaceutical Sciences, Yonsei University, Incheon, Republic of Korea.

**Keywords:** Expenditure Decomposition, Interrupted Time-Series, Multi-indication Drugs, National Healthcare Insurance, Pharmaceutical Expenditure

## Abstract

**Background:**

The increasing utilization of high-cost drugs with multiple indications poses significant financial challenges to healthcare systems worldwide. This study evaluates the financial impact of expanding drug indications in Korea, focusing on pharmaceutical expenditure trend.

**Methods:**

This study analyzed claims data from the National Health Insurance Service (NHIS) to examine drug characteristics and annual expenditure. Interrupted time-series analysis assessed monthly expenditure changes following indication expansions.

**Results:**

We analyzed 57 drugs that expanded their indications between 2012 and 2023. From 2012 to 2022, drug expenditures increased 15-fold (compound annual growth rate [CAGR] 30.8%), a significantly larger rise compared to the 1.9-fold rise (CAGR 6.5%) in total pharmaceutical expenditures covered by the NHIS. Notably, expenditures increased 35-fold for 35 drugs classified under anatomical therapeutic chemical (ATC) code L (antineoplastic and immunomodulating agents) and 375-fold for 26 drugs with risk-sharing agreements (RSAs). Interrupted time-series analysis (n = 27) demonstrated significant monthly expenditure increases before expansion (US$ 0.33 million per month, *P*=.000). There were significant increases in expenditure between the pre- and post-expansion period (US$ 4.99–5.64 million, *P*=.000). Moreover, post-expansion trends showed significant additional increases in expenditure: US$ 0.13 million per month (*P*=.003) at +24 months and US$ 0.07 million per month (*P*=.037) at +36 months.

**Conclusion:**

Despite price reduction strategies for multi-indication drugs, expenditure accelerated increase in expenditure post-expansion of indication. This highlights the need for robust post-pricing management for listed drugs. In the long term, a total budget system could ensure predictable and sustainable financing by providing clear financial boundaries within the health insurance budget.

## Background

**Key Messages**

**Implications for policy makers**

- This study provides an empirical and comprehensive evaluation of the financial impact of expanding drug indications in Korea, utilizing claims data from the National Health Insurance Service (NHIS) to analyze pharmaceutical expenditure trends and policy implications.
- Over the decade, the expenditure on drugs with expanded indications increased significantly compared to the total expenditure on all NHIS-covered pharmaceuticals.
- Expenditure rose notably after expansion, with post-expansion trends showing further significant increases.
- Findings highlight the limitations of current price reduction strategies, the need for robust post-pricing management strategies, and the potential adoption of total budget systems to manage pharmaceutical costs effectively.

**Implications for the public**

Our research highlights the financial burden that expanding drug indications place on Korea’s healthcare system. Analyzing National Health Insurance Service (NHIS) data, we found drug expenditures increased by over 15-fold between 2012 and 2023, particularly for costly cancer and immunotherapy drugs. Following expansion, expenditure showed a significant initial rise, followed by continued increases over time. Despite price reduction efforts, expenditures continued to climb. Our findings suggest current pricing strategies are insufficient although multi-indication drugs, which are pharmaceuticals approved for multiple therapeutic uses, provide broader treatment options to patients. We recommend stronger post-pricing management and total budget systems to ensure healthcare remains affordable while maintaining access to essential treatments.

With the rise of specialized therapies, one pharmaceutical product targeting fundamental biological processes or pathways emerges as useful for multiple indications.^1^ For instance, targeted immunotherapies released over the past 25 years have an average of four indications.^2^ Approximately one-fourth of the new solid tumor therapies approved in the United States between 2011 and 2021 received subsequent approvals for additional indications.^3,4^ Additionally, 34% of all blood disorder treatments received approval for indications other than their initial approvals in the United States.^2^ South Korea (hereafter Korea) has also continuously expanded the usage scope for some high-cost drugs (such as immune-oncology agents). Out of the 14 new drugs introduced in 2017 under the risk sharing agreement (RSA), also called managed entry agreement, ten (or approximately 71%) have undergone an expansion in their usage scope in Korea.^5^

 Multi-indication drugs, which are pharmaceuticals approved for multiple therapeutic uses, are important in research, given their potential to provide broader treatment options, economic implications, and regulatory challenges.^6^ In the case of multi-indication drugs, pharmaceutical companies have more information on their products, and thus, they are likely to choose reimbursement strategies such as the order of indications for market access submission to maximize revenue. At the same time, insurers find it challenging to respond effectively despite information asymmetry for sustainable insurance budget management.^7,8^

 In Korea, drugs seeking market access for indication expansion must undergo a mandatory health technology assessment (HTA) against alternative drugs if they already received an evaluation for their initial indication, especially among RSA drugs. Therefore, firms have an incentive initially to list an indication for a small market (with a minimal budget impact on the insurer). They then expand indications to larger markets, even if the economic evaluation data is not impressive. This approach could potentially introduce financial challenges to the insurer.^9^

 As drug costs gradually increase compared to the expected claim amounts,^10^ there is a growing need for rigorous ex-post management after the initial listing of pharmaceuticals.^11,12^ However, no comprehensive empirical research estimates the budget impact of drugs with expanded indications on a broader real-world scale. Previous research has primarily focused on the status or the budget impact of a limited number of high-cost drugs through case studies or scenario analyses.^13-15^ Additionally, the impact of a drug reimbursement policy can vary by regional characteristics such as regulatory processes, healthcare technology assessment systems, and socio-economic demographic factors.^16^ Therefore, comprehensive evaluations are crucial for understanding the financial implications and developing strategies for effective insurance management.

 This study evaluates the impact of expanded drug indication scopes on the utilization of target drugs. Through this evaluation, we seek to derive implications for effective insurance management in response to the growing use of drugs with multiple indications.

## Methods

###  Policy Background

 Since 1989, Korea has implemented its National Health Insurance Service (NHIS) nationwide, ensuring coverage for the entire population. Therefore, insurance coverage for pharmaceuticals is crucial in Korea. Once pharmaceuticals receive approval for sale from the Ministry of Food and Drug Safety, the insurer gradually determines their reimbursement status and pricing.^17^ Notably, starting in December 2013, Korea applied the RSA to high-priced pharmaceuticals among newly approved drugs to enhance accessibility.^18^ RSAs aim to balance patient access to high-cost drugs while managing financial risks for insurers. Particularly, Korea can apply the RSA to drugs used for severe conditions, such as those without alternatives for cancer treatment or drugs for rare diseases.^19,20^

 When the scope of drug usage expands (including widening eligibility criteria, extending administration periods, and expanding the target population) from the existing reimbursement, the insurer adjusts the listing price for the drug. Before 2014, pharmaceutical companies voluntarily made price reductions agreed upon by the Ministry of Health. However, a dual system has been in operation since 2014. When expecting additional expenditure to be less than approximately US$ 7 million and the drug is not under an RSA, authorities adjust the listing price by a predetermined rate based on additional expected expenditure and the rate of the expected increase. Otherwise, the firm and the insurer proceed with a drug price negotiation.^21^ In the negotiation pathway, authorities also reset the RSA if it applied previously. The factors influencing negotiated prices include clinical value, cost-effectiveness, international reference pricing, availability of alternative treatments, budget impact, and previous price reduction history, among others.^22^

###  Data

 This study used drug indication expansion information and claims data from the NHIS, and drug approval details from the Ministry of Food and Drug Safety. We obtained the list and details of drugs with expanded indications from the NHIS and the Health Insurance Review and Assessment Service (HIRA), the administrative agencies responsible for managing these drugs. We identified drugs that expanded their indication scope between 2017 and 2023.

**Figure 1:**
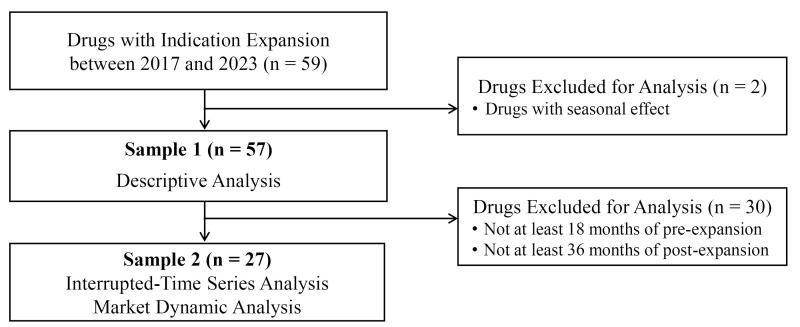
Selection Flow of Drugs for Analysis. *Note.* We used Sample 1 for descriptive analysis and Sample 2 to analyze the impact of drug indication expansion on expenditure and the decomposition of relative change in expenditure between pre-expansion and post-expansion periods.

 We applied exclusion criteria for the analysis as follows: (1) drugs with seasonal effects (eg, oseltamivir), and (2) drugs without sufficient pre- or post-expansion time for analysis. A diagram detailing the sample selection flow is in Figure 1.

 Sample 1 includes 57 drugs with indication expansion between 2017 and 2023 after excluding two drugs with seasonal effects. Sample 2 consists of 27 drugs selected from Sample 1 for interrupted time series and market dynamic analyses. The data period spans between 2012 and 2022. We used Sample 1 for descriptive analysis and Sample 2 to analyze the impact of drug indication expansion on expenditure and the decomposition of relative change in expenditure between pre-expansion and post-expansion periods.

 The dataset covers claims data of drugs of interest from the NHIS, spanning January 2012 to December 2022. We collected details about these drugs, including their initial date of reimbursement, the number and dates of indication expansions, anatomical therapeutic chemical (ATC) code, presence of RSAs or negotiations, orphan drug status, pivotal clinical trials, and market approval conditions, aiming for a comprehensive understanding of their profiles.

 The main outcome variable in this study is the monthly expenditure for each drug, which includes costs subsidized by the NHIS and out-of-pocket expenses incurred by individuals.

###  Analysis

 We conducted a descriptive analysis to summarize the characteristics of drugs with indication expansion (Figure 1, Sample 1). We then conducted an interrupted time-series analysis to assess the impact of drug indication expansion on monthly drug expenditures for 27 of the 57 analyzed drugs (Figure 1, Sample 2). We selected these 27 drugs as they had at least 18 months of follow-up before and 36 months of follow-up after the indication expansion.

 We performed an interrupted time-series analysis based on follow-up periods of 12, 24, and 36 months after indication expansion, comparing them to the pre-expansion period of 18 months. The expansion timing varies for each drug. Figure S1 presents the study framework. Throughout the analysis, we categorized and examined the drugs separately based on whether they were under RSAs or classified under ATC code L (antineoplastic and immunomodulating agents).

 Equation (1) defines the interrupted time-series analysis model for drug expenditure.^23^


(1) $$Y_{t}=\beta_{0}+\beta_{1}\times TIME_{t}+\beta_{2}\times POLICY_{t}+\beta_{3}\times POLICY_{t}\times TIME_{t}+\varepsilon_{t}$$

Here, Y_t_ represents the monthly expenditure of drugs with expanded indication at time *t*. POLICY_t_ represents the first expansion of drug indication, and TIME_t_ is the time since the start of the study. In our study, the expansion timing varied depending on each drug analyzed. Therefore, we set TIME_t_ to 0 in the initial month of the first drug in expansion, taking negative and positive values for months before and after the first expansion, respectively (eg, TIME_t_ = 10 for the tenth month after expansion). *β*_1_ signifies the slope or trajectory of the drug expenditure until the introduction of the first expansion. *β*_2_ represents the average difference in expenditures between the pre- and post-expansion periods. *β*_3_ indicates the difference in the slopes of expenditure between pre- and post-expansion. Significant *β*_2_ indicates an immediate expansion effect, while *β*_3_ indicates an expansion effect over time. We performed and presented the interrupted time-series analysis by group and follow-up period: total, ATC code L versus other, and RSA versus non-RSA in 12 months following the first indication expansion, 24 months, and 36 months.

 Additionally, we conducted a market dynamic analysis by decomposing the growth of drug expenditure ex-post expansion compared to before expansion into three components: price (unit drug cost), quantity (sales volume), and case mix (composition of drugs in the market) (Figure 1, Sample 2). Equations (2) and (3) show the decomposition of the total drug expenditure into the price, exposure, and case mix.


(2) $$C=\sum_{i=1}^{n}p_{i}\times q_{i}$$

(3) $$\begin{array}{l} \frac{C_{1}}{C_{0}}=\sum_{i=1}^{n}\frac{p^{1}q^{1}}{p^{0}q^{0}}=\frac{\sum_{i=1}^{n}q^{1}\times \frac{\sum_{i=1}^{n}p^{1}q^{1}}{\sum_{i=1}^{n}q^{1}}}{\sum_{i=1}^{n}q^{0}\times \frac{\sum_{i=1}^{n}p^{0}q^{0}}{\sum_{i=1}^{n}q^{0}}}=\\ \frac{\sum_{i=1}^{n}q^{1}}{\sum_{i=1}^{n}q^{0}}\times \frac{\sum_{i=1}^{n}p^{1}q^{0}}{\sum_{i=1}^{n}p^{0}q^{0}}\times \frac{\frac{\sum_{i=1}^{n}p^{1}q^{1}}{\sum_{i=1}^{n}q^{1}}}{\frac{\sum_{i=1}^{n}p^{1}q^{0}}{\sum_{i=1}^{n}q^{0}}}=I^{P}\times I^{Q}\times I^{U} \end{array}$$

where *i* is the drug, I^Q^ is the exposure index, I^P^ is the price index, and I^U^ is the case mix index. C, p, and q represent total expenditure, volume-weighted price, and volume, respectively. The subscripts 0 and 1 indicate pre- and post-expansion periods, respectively.

 The decomposition shows the individual contributions of price, quantity, and composition to total drug expenditure pre- and post-expansion.^24^ The indication expansion naturally increases sales volume given the additional indication to the existing indication, whereas the listing price generally decreases at expansion. Therefore, we assumed that sales volume for those drugs exceeds the pre-expansion magnitude, and the contributions of price and consumption quantity to total drug expenditure would be the opposite for the drugs after the indication expansion. We calculated the relative change in total drug expenditure between the pre- and post-expansion by allowing changes in only one component at a time between periods. The product of the three relative changes between the pre- and post-expansion shows the change in total expenditure between the two periods.^25^ We used SAS (version 9.4; SAS Institute, Cary, NC, USA) for data processing and STATA (version 17) for analysis.

## Results

###  Descriptive Analysis

Table 1 summarizes the characteristics of drugs with the expanded usage scope. Out of 57 drugs, 35 (61.4%) were the ATC code L, 26 (45.6%) had RSAs, and 23 (40.4%) overlapped between ATC L and RSAs. Most (77.2%) of these drugs had initial listings after 2015.

**Table 1:** Characteristics of Drugs With Indication Expansion

	**Total**	**ATC Code**		**Risk Sharing Agreement**	
		**L**	**Others**	**Yes**	**No**
N (%)	57 (100%)	35 (100%)	22 (100%)	26 (100%)	31 (100%)
**Year of Initial Reimbursement**					
Pre-2011	6 (11%)	2 (6%)	4 (18%)	0 (0%)	6 (19%)
2011	1 (2%)	1 (3%)	0 (0%)	0 (0%)	1 (3%)
2013	3 (5%)	0 (0%)	3 (14%)	0 (0%)	3 (10%)
2014	1 (2%)	1 (3%)	0 (0%)	1 (4%)	0 (0%)
2015	2 (4%)	1 (3%)	1 (5%)	1 (4%)	1 (3%)
2016	17 (30%)	11 (31%)	6 (27%)	5 (19%)	12 (39%)
2017	14 (25%)	11 (31%)	3 (14%)	11 (41%)	3 (10%)
2018	4 (7%)	2 (6%)	2 (9%)	1 (4%)	3 (10%)
2019	4 (7%)	3 (9%)	1 (5%)	2 (8%)	2 (6%)
2020	4 (7%)	2 (6%)	2 (9%)	4 (15%)	0 (0%)
2021	1 (2%)	1 (3%)	0 (0%)	1 (4%)	0 (0%)
**Year of Indication Expansion**					
2017	3 (5%)	2 (6%)	1 (5%)	2 (8%)	1 (3%)
2018	13 (23%)	10 (29%)	3 (14%)	8 (31%)	5 (16%)
2019	18 (32%)	6 (17%)	12 (55%)	5 (19%)	13 (42%)
2020	7 (12%)	5 (14%)	2 (9%)	3 (12%)	4 (13%)
2021	11 (19%)	8 (23%)	3 (14%)	4 (15%)	7 (23%)
2022	4 (7%)	4 (11%)	0 (0%)	3 (12%)	1 (3%)
2023	1 (2%)	0 (0%)	1 (5%)	1 (4%)	0 (0%)
**Gap Between Initial Reimbursement and Indication Expansion (Months)**					
Mean, SD	49.0, 51.8	41.0, 49.1	61.8, 54.6	22.9, 13.1	70.9, 61.5
Min, Max	2.0, 265.8	5.5, 265.8	2.0, 200.9	5.5, 59.8	2.0, 265.8
**Number of Indication Expansions**					
1	46 (81%)	26 (74%)	20 (91%)	19 (73%)	27 (87%)
≥2	11 (19%)	9 (26%)	2 (9%)	7 (27%)	4 (13%)
**Price Reduction at First Indication Expansion (%)**					
Mean ± SD	4.4 ± 4.7	4.9 ± 5.4	3.5 ± 3.1	5.7 ± 6.2	3.3 ± 2.5
Min, Max	0, 23.1	0, 23.1	0, 11.9	0, 23.1	0, 11.9
**Negotiation**					
Yes	31 (54%)	24 (69%)	7 (32%)	25 (96%)	6 (19%)
No	26 (46%)	11 (31%)	15 (68%)	1 (4%)	25 (81%)
**Orphan Drug**					
Yes	15 (26%)	12 (34%)	3 (14%)	14 (54%)	1 (3%)
No	42 (74%)	23 (66%)	19 (86%)	12 (46%)	30 (97%)

Abbreviations: SD, standard deviation; ATC, anatomical therapeutic chemical; L, antineoplastic and immunomodulating agents.

 The first indication expansion occurred between 2017 to 2023, with an average gap of 49.0 months (standard deviation [SD] of 51.8 months, with a minimum of 2.0 months, and a maximum of 265.8 months) between the initial listing and expansion dates. During the analysis period, 80.7% of drugs expanded their usage scope once, while others did so two or three times. When the company added a new indication to the listed drugs, their price decreased by an average of 4.4% (SD 4.7%, minimum 0%, maximum 23.1%). Supplementary file 1 (Table S1) shows the drugs included in the analysis.

 The expenditure of total pharmaceuticals covered by the NHIS increased from approximately US$ 9.3 billion in 2012 to about US$ 17.2 billion by 2022, representing a 1.8-fold increase with a compound annual growth rate (CAGR) of 6.5%. Meanwhile, the number of pharmaceuticals based on active ingredients remained relatively stable at around 3000, whereas the number of products increased a 1.6-fold rising from 15 620 to 25 254 over the same period (Figure S2).

**Figure 2:**
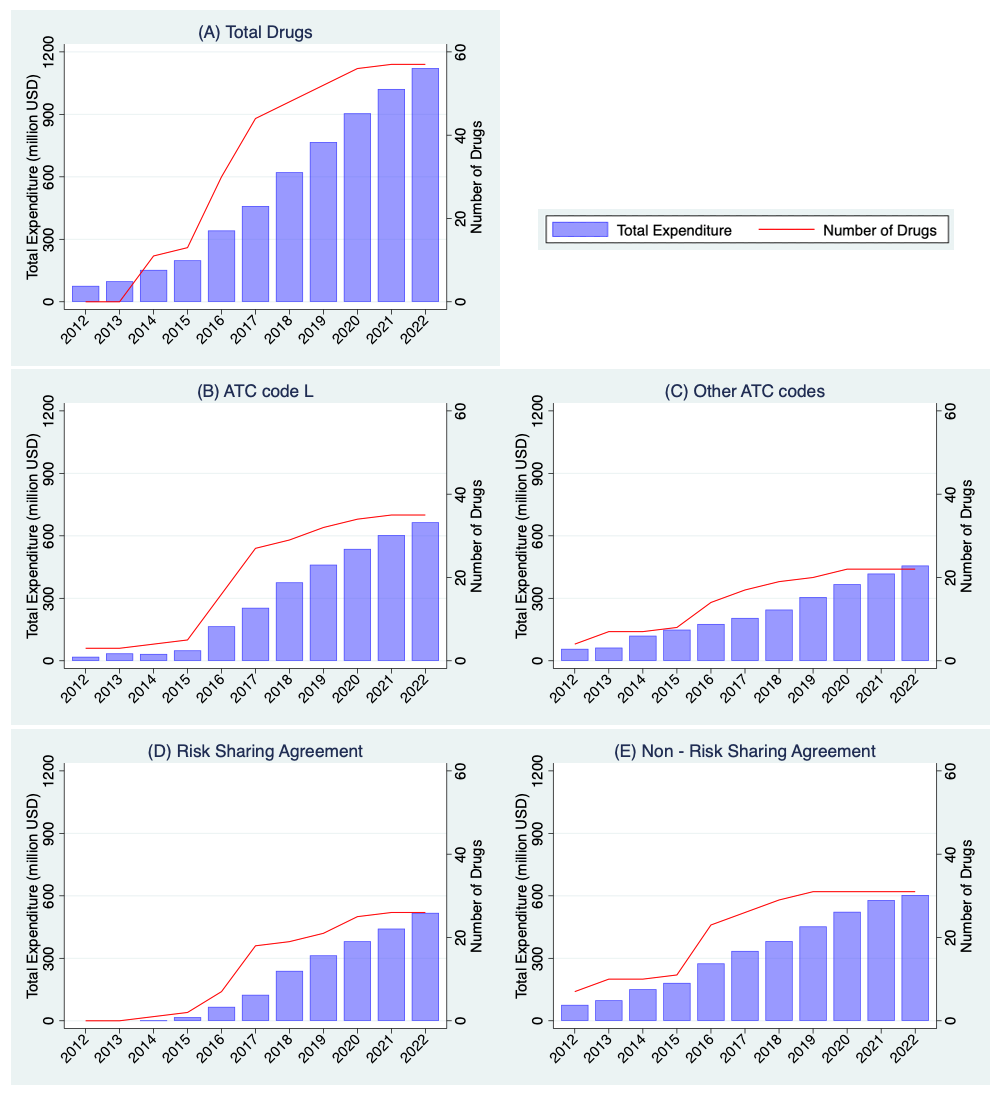
Annual Expenditure Changes of Drugs With Indication Expansion. (A) total drugs, (B) anatomic therapeutic chemical (ATC) code L, (C) other ATC Codes. (D) risk sharing agreement, and (E) non-risk sharing agreement. Abbreviations: ATC, anatomical therapeutic chemical; L, antineoplastic and immunomodulating agents.

Figure 2 illustrates the annual expenditure change for the analyzed drugs, showing a substantial increase from 2012 to 2022. Total drug expenditure increased 15-fold, from approximately US$ 76.3 million in 2012 to about US$ 1122 million in 2022. Specifically, expenditure for drugs classified under the ATC L code (ie, anticancer drugs) increased 35-fold, while those with RSAs saw a staggering 375-fold rise since their introduction in 2014. In 2022, drugs categorized under the ATC L code and those with RSAs accounted for 58.1% and 49.9% of all drugs, respectively. The CAGR of total expenditure over 10 years was 30.8%, with 42.7% for drugs under the ATC L code and 80.9% for those under RSAs since 2014.

 Regarding the number of drugs involved in expenditure during the same period, there was a substantial eight-fold increase, from seven in 2012 to 57 in 2023. ATC L code drugs increased 12-fold, and those with RSAs rose 26-fold. However, the growth in the number of drugs was lower than the increase in expenditure.

###  Interrupted Time-Series Analysis on the Impact of Expanding Drug Indication

**Figure 3:**
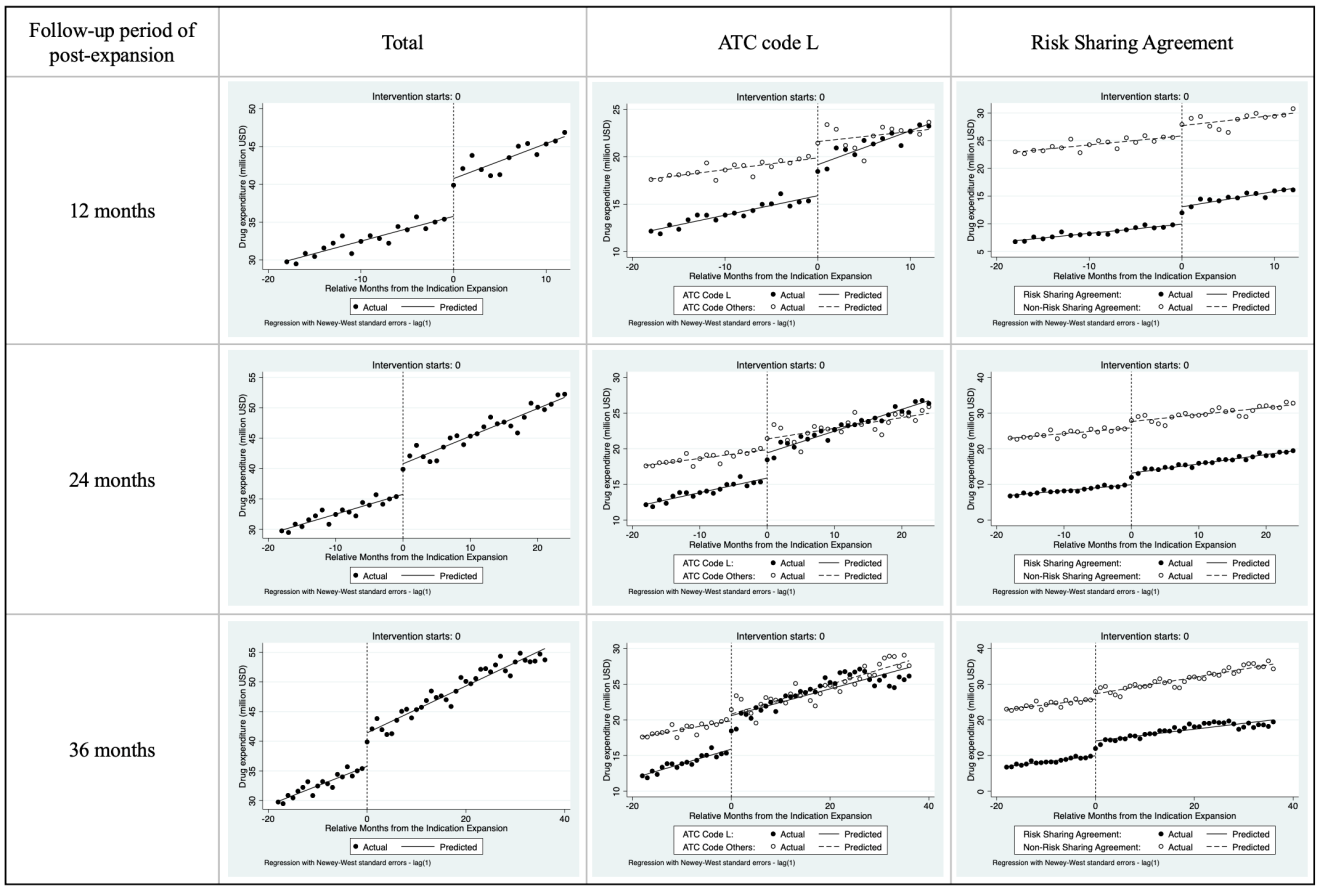
Interrupted Time-Series Analysis of Expenditure for Drugs With Indication Expansion. Note. Dash line (month = 0) indicates the first month when the indication of drugs was expanded after they were initially listed in National Health Insurance. Sample 2 (n = 27) was used to conduct the interrupted time-series analysis, including 12 drugs with ATC code L and 10 drugs with RSA.

 We conducted an interrupted time-series analysis to assess the impact of expanding drug indications on monthly drug expenditure for 27 drugs across different groups and follow-up periods post-expansion (refer to Figure 3 and Table 2).

**Table 2:** Interrupted Time-Series Analysis of Expenditures for Drugs With Indication Expansion

**Follow-up Period of Post-expansion**	**Variables**	**β Estimates (SE)**				
		**Total**	**ATC Code**		**Risk Sharing Agreement**	
			**L**	**Others**	**Yes**	**No**
12 months	Time trend pre-expansion monthly (*β*_1_)^a^	0.33^***^ (0.02)	0.20^***^ (0.02)	0.12^***^ (0.01)	0.17^***^ (0.01)	0.16^***^ (0.01)
	Indication expansion (*β*_2_)^b^	4.99^***^ (0.77)	3.27^***^ (0.46)	1.72^*^ (0.74)	3.13^***^ (0.45)	1.86^*^ (0.70)
	Difference of time trend between pre-and post-expansion (*β*_3_)^c^	0.14 (0.08)	0.15^*^ (0.06)	-0.02 (0.07)	0.11^*^ (0.05)	0.02 (0.07)
24 months	Time trend pre-expansion monthly (*β*_1_)^a^	0.33^***^ (0.02)	0.20^***^ (0.02)	0.12^***^ (0.01)	0.17^***^ (0.01)	0.16^***^ (0.01)
	Indication expansion (*β*_2_)^b^	5.02^***^ (0.58)	3.52^***^ (0.36)	1.50^**^ (0.58)	3.24^***^ (0.30)	1.78^**^ (0.52)
	Difference of time trend between pre-and post-expansion (*β*_3_)^c^	0.13^**^ (0.04)	0.10^**^ (0.03)	0.03 (0.04)	0.09^***^ (0.02)	0.03 (0.03)
36 months	Time trend pre-expansion monthly (*β*_1_)^a^	0.33^***^ (0.02)	0.20^***^ (0.02)	0.12^***^ (0.01)	0.17^***^ (0.01)	0.16^***^ (0.01)
	Indication expansion (*β*_2_)^b^	5.64^***^ (0.52)	4.70^***^ (0.51)	0.95 (0.51)	4.19*** (0.41)	1.45^**^ (0.45)
	Difference of time trend between pre-and post-expansion (*β*_3_)^c^	0.07^*^ (0.03)	-0.02 (0.03)	0.08^**^ (0.02)	0.00 (0.02)	0.07^**^ (0.02)

Abbreviations: ATC, anatomical therapeutic chemical; L, antineoplastic and immunomodulating agents; SE, standard error. *Note*. We measured expenditure in million USD: (a) Monthly expenditure on average before indication expansion of drugs, (b) The difference in expenditure between pre-and post-expansion, and (c) The difference in the slopes of expenditure between pre-and post-expansion; *** *P* < .001, ** *P* < .0.01, * *P* < . 05.

Figure 3 illustrates the monthly expenditure trends before and after the indication expansion of these 27 drugs, including 12 drugs with ATC code L and ten drugs with RSA. The figure shows a steady and additional increase in expenditure post-expansion, with a more noticeable rise in drugs with ATC code L.

 In Table 2, all drugs showed a significant monthly average expenditure increase of US$ 0.33 million before the expansion (*β*_1_, *P* = .000, 95% CI 0.28, 0.37). This trend was consistent across groups such as ATC code L (US$ 0.20 million, *P* = .000) and other ATC codes (US$ 0.12 million, *P* = .000), as well as drugs with RSAs (US$ 0.17 million, *P* = .000) and those without RSAs (US$ 0.16 million, *P* = .000). Following expansion, total average expenditure significantly increased by US$ 4.99 to 5.64 million (*β*_2_, *P* = .000), varying slightly across different post-expansion analysis periods. We consistently observed this significant increase within each group: ATC code L (US$ 3.27–4.7 million) and other ATC codes (US$ 0.95–1.72 million), as well as drugs with RSAs (US$ 3.13–4.19 million) and those without RSAs (US$ 1.45–1.86 million).

 The impact of expansion on post-expansion trends (*β*_3_) showed variability across analysis periods. In the 12 months following the expansion, *β*_3_ was insignificant, indicating no further increase beyond the pre-expansion trend. However, in the 24- and 36-months following expansion, we found a significant further increase in expenditure for all drugs: an average of US$ 0.13 million monthly during the following 24 months (*P* = .003) and an average of US$ 0.07 million monthly during the following 36 months (*P* = .037). This finding suggests that despite introducing price reductions for drugs with expanded indications, the expenditure increase accelerated more than before the expansion. Specifically, in the 12- and 24-month post-expansion analyses, the ATC code L and RSA groups showed a significant additional increase in expenditure. Additionally, the ATC other group and no-RSA groups exhibited significant additional increases in expenditure in the 36 months following the expansion.

###  Market Dynamic Analysis: Decomposition of Relative Change in Total Drug Expenditure

 In Table 3, we show relative changes between the pre- and post-periods in total drug expenditure that we could attribute to changes in a specific expenditure component while holding all other components constant. Holding the price steady would have decreased the total drug expenditure by 14%, whereas volume increase alone would have increased the expenditure by 2.68 times. The decomposition confirms that the volume for those drugs exceeds the pre-expansion magnitude, and the contributions of price and consumption quantity to total drug expenditure would be the opposite for the drugs after the indication expansion.

**Table 3:** Decomposition of Relative Change in Total Expenditure Between Pre-expansion and Post-expansion Periods

**Group**		**Total Expenditure Increase Post-expansion Versus Pre-expansion**	**Exposure Index**	**Price Index**	**Case Mix Index**
Total		2.006	2.680	0.863	0.867
ATC code	L	3.264	3.659	0.908	0.982
	Others	1.911	2.667	0.830	0.864
RSA	Yes	3.637	3.565	0.865	1.179
	No	1.991	2.670	0.863	0.864

Abbreviations: RSA, risk-sharing agreement; ATC, anatomical therapeutic chemical; L, antineoplastic and immunomodulating agents.

## Discussion

 As drugs expand their indications, they can treat a wider range of diseases or symptoms, providing patients with more treatment options and potentially benefiting those who previously had limited choices. However, this expansion can impose a financial burden on the insurer. Our study is the first to empirically assess how indication expansion in insurance coverage affects the usage of target drugs. Our results show the financial impact of additional indications on total expenditure in the real world. Specifically, following indication expansion, spending increased by an average of approximately 7.0 billion KRW, with further increases post-expansion compared to the pre-expansion rate. Although the NHIS reduces the listing through price negotiations for drugs when expanding indications under existing reimbursement policies, our findings suggest that more comprehensive management scheme is needed for the financial sustainability of the NHIS accompanied with improved access by indication expansion.

 Whether indication expansion increases total pharmaceutical expenditure when accompanied by price reductions remains an empirical question, particularly under a single price scheme for all indications. If the increase in drug usage after an indication expansion is not large enough to offset the price reduction, the total expenditure may not necessarily increase. However, if the first indication has a small market size and the expanded indication has a much larger patient population, total expenditure could increase significantly, even with a uniform price reduction. Additionally, rising disease prevalence could further drive up spending. Due to data limitations, we did not isolate the impact of prevalence changes for each indication. However, we found that the increase in the time trend of total drug expenditure, including single-indication drugs that have not undergone indication expansion, was much slower compared to the steeper growth observed in drugs with indication expansion (CAGR of 6.5% vs. 30.8% over a decade). Additionally, the total volume of sample molecules, used as a proxy for prevalence, contributed the most to this expenditure growth, reinforcing the link between indication expansion and rising costs.

 The global data illustrates the variations in drug prices by the sequence of indications and countries for multi-indication cancer drugs approved in the United States. Germany and France experienced a price decline, whereas the United Kingdom and Canada maintained stable prices without notable fluctuations.^26^ However, there was a distinct upward trend in drug prices in the United States compared to the initial indication.^27^ One study based on Korean data identified 54 targeted therapy and immune-oncology drugs reimbursed domestically, of which 32 had multiple indications. Price decreased by 7.38% on average for 26 of these drugs after expanding reimbursement criteria. The magnitude of the price reductions was larger as the expansion frequency rose.^14^ Our study builds on this research by assessing how formal indication expansion under Korea’s reimbursement scheme affects total drug expenditure across all therapeutic areas. We found that oncology drugs experienced the largest increase in expenditure, indicating that price reductions under a single-price-per-molecule system did not prevent total spending from rising as drug usage increased.

 Most global healthcare systems, including Korea, assign a single price to a drug regardless of its number of indications.^28^ Under this system, pharmaceutical companies often seek reimbursement first for indications with the highest clinical efficacy, which typically apply to well-defined, smaller patient populations.^29,30^ This strategy allows firms to generate real-world evidence, establish a favorable pricing benchmark, and strengthen future negotiations for additional indications.^2^

 Previous studies have corroborated firms’ strategies to launch indications with the highest value first to anchor a favorable listing price. Among the 100 Food and Drug Administration (FDA)-approved multi-indication anticancer drugs, 25 were for “primary indications,” while 75 fell under “additional indications.” Initial indications were more likely than additional indications to receive conditional approvals (30.2% vs. 14.2%), orphan drug designations (43.8% vs. 22.2%), and priority reviews (12.6% vs. 9.8%) in all four regulatory agencies, including the FDA, European Medicines Agency, Health Canada, and Australia Therapeutic Goods Administration.^27^ Similarly, the initial reimbursed indications covered smaller patient populations and demonstrated greater clinical benefits than later-approved indications.^26^ A study in Italy found that second-round negotiations for expanded indications took longer and resulted in lower rebate rates than initial negotiations. This finding suggests that pharmaceutical companies prioritize securing higher prices for initial listings before negotiating for lower-value indications, potentially to maximize profits.^2,31^

 Globally, the median time between first and second indication approvals is 1.7 years, with a median of 0.65 new indications approved per year.^2^ However, our data showed a much longer lag between initial listing and first expansion in Korea, averaging 49.0 months (ranging from 2 to 265.8 months). This finding implies that pharmaceutical launch strategies vary depending on a county’s regulatory context. For instance, Korea’s NHIS imposes a single fixed price for multi-indication drugs without adjusting for usage differences across indications. Therefore, pharmaceutical companies are likely to be more cautious in their launch strategy, as they aim to minimize price reductions and potential revenue losses.^14^

 We did not directly isolate the incremental increases in drug expenditures associated with indication expansion. Although reimbursement claims specify the primary indication for treatment, the Korean system assigns a single price and reimbursement code to each molecule, regardless of its intended use. Furthermore, some indication expansions involve different lines of therapy within the same disease (eg, first- vs. second-line therapies for cancer treatments) rather than distinct therapeutic areas or different diseases within a therapeutic area. Due to these complexities, we did not present the number of indications per drug in our analysis. Furthermore, our analysis relied on list prices, which may differ from actual transaction prices for RSA drugs due to confidentiality agreement. While this could lead to overestimating absolute expenditure for RSA drugs, it is unlikely to have a significant impact on the trend (slope) of expenditure growth. Thus, our findings regarding the impact of indication expansion remain valid even with RSA drugs included.

 When drugs receive reimbursement or expand their indication, they can partially replace existing reimbursed treatments for the same conditions. Korea has required HTAs to assess the cost-effectiveness for new drug reimbursement decisions since 2007. However, some drugs, such as those for ultra-rare diseases or life-threatening diseases, are exempt, particularly those approved under conditional or rolling reviews.^17^ If the initial listing received approval without an HTA for the comparative cost-effectiveness, subsequent indications for that drug also bypass the assessment in Korea. Furthermore, while HTA results and supporting evidence for initial reimbursement decision are publicly available on the government website, only the final results are published for expanded indications, without the supporting evidence. Therefore, specifying standard of care treatments the newly reimbursed indications replace is difficult. Also, estimating the quantity replaced for the standard of care by a newly reimbursed indication is challenging due to limited data on patient numbers for multi-indication drugs, which is a key element for budget impact calculation.^13^

 The number of high-cost, multi-indication drugs is increasing,^1^ underscoring the need for long-term management strategies. Each country has a unique approach to regulating new indications for reimbursed drugs, and thus, the actual impact on consumer surplus of setting indication-based pricing depends on the healthcare system’s structure.^6,32^ In Korea, where NHIS operates as a single-payer, and patients bear the out-of-pocket costs through co-insurance, indication-based pricing may not necessarily benefit consumers, particularly if value-based prices exceed production costs.^33^ Product surplus optimization might enhance total social welfare but in cost of reduced or demolished consumer surplus.^29^ Beyond consumer and social welfare considerations, implementing an indication-based pricing system would require institutional reforms and broad public consensus. The additional administrative costs and complexity of tracking indication-specific values against potential benefits should be weighed.

## Conclusions

 Due to societal demands for high-cost drugs (such as immuno-oncology drugs), the scope of use is continuously expanding, and the actual drug costs are gradually increasing compared to the expected amounts at the time of listing. This highlights the need for thorough post-pricing management for listed drugs. In the long term, a total budget system with definite e “total budget” level, could facilitate smoother negotiations between pharmaceutical companies and insurers, including during indication-expansion negotiations, by providing clear financial boundaries based on the health insurance budget.

## Ethical issues

 This study was approved by the Institutional Review Board of Yonsei University (IRB No. 202303-HR-3269-01).

## Conflicts of interest

 EH reports financial support was provided by NHIS. All the other authors declare that they have no known competing financial interests or personal relationships that could have appeared to influence the work reported in this paper.

## Data availability statement

 The datasets generated and/or analyzed during the current study are not publicly available due to restrictions from the National Health Insurance, but are available from the corresponding author on reasonable request.

## Supplementary files

Supplementary file 1 contains Table S1 and Figures S1-S2.
