# Supplementary material for: Assessment of Financial Impact of Expanding the Scope of Drug Usage in South Korea
Source: Int J Health Policy Manag. 2025 Jul 13;14:8812. doi: 10.34172/ijhpm.8812 (PMC12414146; doi:10.34172/ijhpm.8812)
Supplement: Supplementary file 1 — contains Table S1 and Figures S1-S2. [file ijhpm-14-8812-s001.pdf]

Article title: Assessment of Financial Impact of Expanding the Scope of Drug Usage in South Korea

Journal name: International Journal of Health Policy and Management (IJHPM)

Authors' information: Su-Yeon Yu[1], Dong-Sook Kim[2], Hyungmin Kim[3,4], Junwoo Jo[5], Hyunduck Kim[3], Euna Han[6]*

[1]College of Pharmacy, Kangwon National University, Chuncheon, Republic of Korea.

[2]Department of Health Administration, College of Nursing and Health, Kongju National University, Kongju, Republic of Korea.

[3]Department of Drug Management, National Health Insurance Service, Wonju, Republic of Korea.

[4]College of Pharmacy, Seoul National University, Seoul, Republic of Korea.

[5]Department of Statistics, Kyungpook National University, Daegu, Republic of Korea.

[6]College of Pharmacy, Yonsei Institute of Pharmaceutical Sciences, Yonsei University, Incheon, Republic of Korea.

***Correspondence to:** Euna Han; Email: eunahan@yonsei.ac.kr

Citation: Yu SY, Kim DS, Kim H, Jo J, Kim H, Han E. Assessment of financial impact of expanding the scope of drug usage in South Korea. Int J Health Policy Manag. 2025;14:8812. doi:10.34172/ijhpm.8812

Supplementary file 1

Table S1: List of Ingredients in Analyzed Drugs

Abiraterone Acetate
Alectinib Hydrochloride (as Alectinib)
Atezolizumab
Atomoxetine 1
Atomoxetine 2
Bedaquiline Fumarate (as Bedaquiline)
Belimumab
Bevacizumab
Blinatumomab
Capecitabine
Celecoxib
Cetuximab
Clostridium Botulinum A Toxin
Clostridium Botulinum Toxin Type A Haemagglutinin Complex

Crizotinib

Dabrafenib Mesylate (as Dabrafenib)

Defibrotide

Denosumab 1

Denosumab 2

Dupilumab

Eculizumab

Emicizumab

Enzalutamide

Everolimus

Fulvestrant

Human-Derived Epidermal Keratinocyte

Ibrutinib Inotuzumab

Ozogamicin

Iron Hydroxide Sucrose Complex

Lenvatinib

Methylphenidate

Niraparib Tosylate (as Niraparib)

Nivolumab

Olaparib

Oxaliplatin

Paclitaxel (Albumin-Bound)

Palbociclib

Pembrolizumab

Pemetrexed 1

Pemetrexed 2

Pertuzumab

Pomalidomide Propofol

Recombinant Blood Coagulation Factor IX, Nonacog A

Recombinant Blood Coagulation Factor VIII

Regorafenib Hydrate (as Regorafenib)
Ribociclib Succinate (as Ribociclib)
Sacubitril/Valsartan
Somatropin Sorafenib
Tegoprazan
Tenofovir Disoproxil Fumarate
Tocilizumab
Tolvaptan Sd Powder (as Tolvaptan)
Trametinib Dimethyl Sulfoxide (as Trametinib)
Trastuzumab Emtansine (as Trastuzumab)
Venetoclax

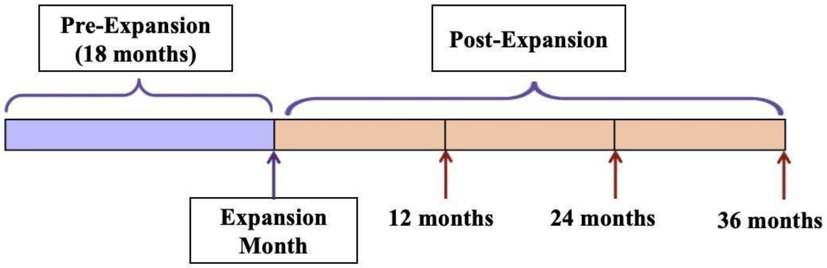

Figure S1. Overview of interrupted time-series analysis.

Note. We performed the interrupted time-series analysis based on follow-up periods of 12, 24, and 36 months after indication expansion, comparing them to the pre-expansion period of 18 months. The expansion timing varies for each drug.

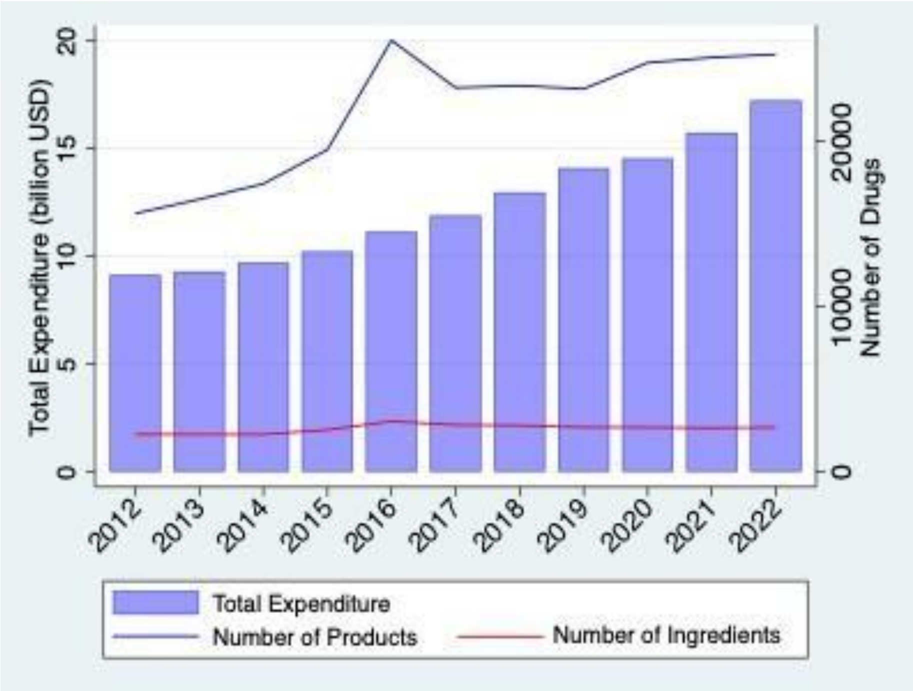

Figure S2. Annual Trends of total pharmaceuticals covered by National Health Insurance. (A) ingredients. (B) products.
